# Synthesis and Biological Evaluation of Novel Alkyl-Imidazolyl Carbinols and their Esters: Potent Antimycotics

**DOI:** 10.3797/scipharm.1304-17

**Published:** 2013-09-02

**Authors:** Jürgen Krauss, Carina Gratzl, Verena Sturm, Christoph Müller, Verena Staudacher, Christoph Q. Schmidt, Franz Bracher

**Affiliations:** Department of Pharmacy – Center for Drug Research, Ludwig-Maximilians University, Butenandtstr. 5–13, 81377 Munich, Germany.

**Keywords:** Grignard reaction, Ergosterol biosynthesis, Sterol biosynthesis inhibitors, Antimycotics

## Abstract

A novel series of imidazol-5-yl carbinols and their 4-chlorobenzoyl esters has been synthesized by the Grignard reaction and subsequent esterification. These compounds were screened for their antimicrobial activities in an agar diffusion assay. The compounds with C_10_ to C_12_-alkyl side chains displayed significant antimycotic activity.

## Introduction

**Fig. 1:**
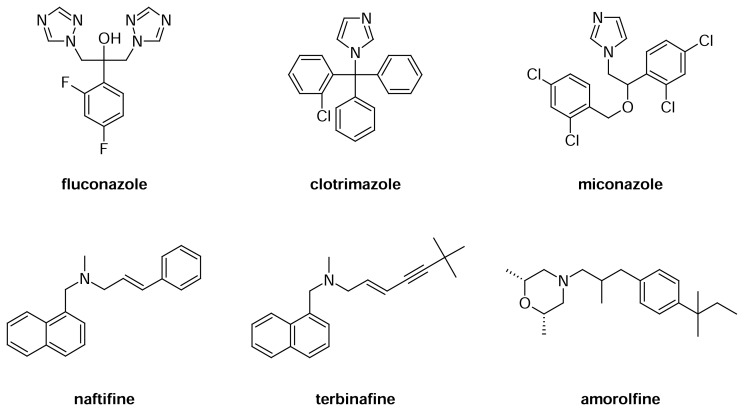
Antimycotic drugs

Ergosterol biosynthesis is an important target for the development of novel antimycotic drugs [1–8]. Three classes of antimycotics, the azoles (*e.g*. fluconazole, clotrimazole, and miconazole), the allylamines (*e.g*. naftifine, terbinafine), and the morpholines (amorolfine) are used in the treatment of human mycoses caused by various species of fungi (Fig. 1). Diverse derivatives of these drug classes are also used in agrochemistry for crop protection or in technical chemistry (*e.g*. as additives in colourings).

These drugs target enzymes in fungal ergosterol biosynthesis. While azoles target C-14 demethylase [7], allylamines inhibit the enzyme squalene epoxidase, and the morpholines inhibit both Δ8,7-isomerase and Δ14-reductase.

Increasing fungal resistance against these antimycotics [2] and their often observed unfavourable pharmacokinetics call for the development of new antimycotics with new targets and better biopharmaceutical properties.

It is well-established that a number of enzymatic transformations in the post-squalene part of ergosterol biosynthesis go through carbocationic intermediates (high-energy intermediates, HEI), e.g. those catalyzed by the enzymes sterol Δ8,7-isomerase, C24-methyltransferase, Δ24,28-reductase, and Δ14-reductase [5]. Inhibition of these enzymes can be achieved effectively by imitation of the respective HEI, since mimics of HEI have a higher affinity for the active site than the substrate sterols have [4]. Synthetic drugs which imitate HEI have to be cationic in the cellular environment, which is most conveniently achieved by the presence of an aliphatic amino group or heteroaromatic ring (which are protonated to a significant extent at physiological pH). The allylamines and the morpholine antifungal amorolfine are the most prominent representatives of this type (Fig. 1). In the class of morpholines and related *N*-alkyl piperidines, the length of the aliphatic or arylaliphatic *N*-substituent significantly determines the potency and selectivity (Δ8,7-isomerase versus Δ14-reductase inhibition) of the compounds [9]. On the other hand, the azole-type antifungals (imidazoles like clotrimazole and miconazole, triazoles like fluconazole, as well as numerous related azoles used in agrochemistry [10]) are inhibitors of the enzyme sterol C14 demethylase. This enzyme, a member of the CYP family, catalyzes the oxidative demethylation of lanosterol, and inhibition is based on the complexation of its heme iron by one of the nitrogen atoms of the unprotonated azole ring. Most of the commercial azole antifungals contain a 1-(2-hydroxyethyl)azole subunit (see fluconazole, Fig. 1), but etherification (miconazole) and esterification [11] of the hydroxyl group is tolerated well. The azoles further inhibit another CYP-enzyme in ergosterol biosynthesis, namely Δ22-desaturase [12]. This enzyme, catalyzing the introduction of an olefinic double bond in the sterol side chain, has been poorly characterized until now, and its selective inhibitors are not yet known. Previously synthesized sterol derivatives containing imidazole groups in the side chain have not inhibited this enzyme, but the sterol C24-methyltransferase has, due to an imitation of a cationic HEI by the protonated imidazole [13].

In the investigations described here, we intended to prepare hybrids of the abovementioned types of established antifungals, aimed at the development of new inhibitors of ergosterol biosynthesis, probably with dual action.

As the central functionality, we selected the imidazole ring (pK_a_ value about 7), which should exist in protonated and unprotonated forms in about equal amounts in cells. In its neutral form it can inhibit the relevant CYP enzymes, whereas the protonated form might imitate carbocationic HEI in sterol biosynthesis [13]. In contrast to established azole antifungals [10], in which the hydroxyalkyl residue is attached to N-1, our target compounds contain side chains (derived from the alkyl chains of morpholine antifungals [9]) at C-5 of the imidazole ring.

## Results and Discussion

### Chemistry

In order to achieve a hydroxyalkylimidazole structural element, we selected a Grignard reaction as the crucial step. Commercially available 1-methyl-1*H*-imidazole-5-carbaldehyde (**1**) was reacted with a series of alkyl and arylalkyl magnesium bromides to give the racemic carbinols **2a**–**g** in moderate-to-good yields. In order to investigate the hitherto only poorly investigated influence of esterification of antifungal hydroxyalkyl-imidazoles [11] and to introduce a chlorophenyl ring (cf. clotrimazole, Fig. 1) into the molecule, selected carbinols (**2b** and **2c**) were esterified with 4-chlorobenzoyl chloride [14] to give the esters **3b** and **3c**. We also intended to achieve etherification of **2a–g** with 2,4-dichlorobenzyl chloride (cf. miconazole, Fig. 1) by the use of a standard protocol with the bases sodium hydride or sodium ethoxide, but unfortunately did not observe any conversion.

### Biological Activity

The antifungal and antibacterial activities of the resulting compounds were determined in an agar diffusion assay against four strains of bacteria (Gram-negative and Gram-positive) and four strains of fungi (two yeasts, mould, dermatophyte) [15]. Except for **2c**, the compounds showed no or only weak activities against the bacteria, when compared to tetracycline, but especially compounds **2c**, **2d**, and **2e**, which contain C_10_ to C_12_-alkyl chains, show remarkable activity against fungi when compared to clotrimazole. The corresponding esters **3b/3c** did not show significant antimicrobial activities. The MIC (minimum inhibitory concentration) against the opportunistic pathogen *Candida glabrata*, which is often associated with resistance to fluconazole therapy [16, 17], was determined to be 25 μg/mL (**2c**), 10 μg/mL (**2d**), and 5 μg/mL (**2e**) (clotrimazole: 2.5 μg/mL) [11].

To determinate the mechanism of action, the carbinols **2c**, **2d**, and **2e** were evaluated in an ergosterol biosynthesis assay [18]. Only **2c** showed a moderate accumulation of episterol (ergosta-7,24(28)-dien-3β-ol) in this assay. This indicates that **2c** is a moderate inhibitor of C5 desaturase, a hitherto poorly characterized, putatively non-heme iron-containing oxidase [19]. But since the even more potent analogues **2d** and **2e** do not show signs of accumulation of episterol, the high antimycotic activity of the imidazolyl carbinols must be due to another mechanism of action.

## Conclusion

The novel alkyl imidazolyl carbinols **2c**, **2d**, and **2e** produced in this study show interesting antifungal activities. The investigation of a series of homologs indicated that the length of the alkyl chain is of critical importance, with an optimum length of C_10_ to C_12_. This parallels findings on *N*-alkylimidazoles, where the same chain length was found to lead to the highest antibacterial and antifungal activity [20], and also on antifungal *N*-alkylmorpholines and -piperidines [9].

Although the main mechanism of action does not seem to be an inhibition of the ergosterol biosynthesis pathway (as initially intended), the pronounced antifungal activity of the compounds **2c**, **2d**, and **2e** warrants them to be the starting point for the development of new antimycotics. The rather simple chemical structures of the novel compounds presented here is particularly interesting as they enable a quick, economical, and effortless synthesis approach to further analogues.

## Experimental

### General

Elemental analyses: Heraeus CHN–Rapid; IR-spectra: Perkin-Elmer FT-IR Paragon 1000; MS: Hewlett Packard MS-Engine; electron ionisation (EI) 70 eV, chemical ionisation (CI) with CH_4_ (300 eV); NMR: Jeol GSX 400 (^1^H: 400 MHz, ^13^C: 100 MHz); melting points: Büchi Melting Point B-540 (not corrected); flash column chromatography (FCC): silica gel 60 (230–400 mesh, E. Merck, Darmstadt); GLC-MS: Shimadzu GC-17 A (carrier: He, oven temperature program: 100–280 °C, 10 °C/min, capillary column: Varian VF-5ms 30 m × 0.25, split injector T = 250 °C, detector T = 260 °C).

### General Procedure 1 (Grignard Reaction)

550 mg (5.0 mmol) of 1-methyl-1*H*-imidazole-5-carbaldehyde (**1**) were dissolved in 20 mL dry THF and 7.5 mmol of the Grignard reagent (solution in 50 mL anhydrous THF, freshly prepared from 7.5 mmol of the corresponding alkyl or arylalkyl bromide, two crystals of iodine and 230 mg (10 mmol) magnesium) were added dropwise. The mixture was stirred for 12 h at room temperature, then quenched with 30 mL of aqueous ammonia buffer pH 9.25, and extracted with ethyl acetate (3 × 30 mL). The combined organic layers were dried over Na_2_SO_4_ and the solvent was evaporated. The residue was purified by flash column chromatography (*n*-hexane/ethyl acetate/dimethyl ethylamine 1:1:0.005).

### General Procedure 2 (Esterification)

About 1.0 mmol of carbinol **2b/2c** was dissolved in 25 mL 1,2-dimethoxyethane and 4-chlorobenzoyl chloride (see amounts below) and 3 mL triethylamine (or pyridine) were added. The mixture was stirred for 12 h at room temperature. Then the solvent was evaporated, the residue dissolved in 25 mL 10% aqueous NaOH solution, and extracted with ethyl acetate (3 × 30 mL). The combined organic layers were dried over Na_2_SO_4_ and the solvent was evaporated. The residue was purified by flash column chromatography (n-hexane/ethyl acetate 1:1).

### 1-(1-Methyl-1H-imidazol-5-yl)hexan-1-ol (2a)

The compound was prepared according “General Procedure 1” from 550 mg (5 mmol) 1-methyl-1*H*-imidazole-5-carbaldehyde (**1**) and the Grignard reagent prepared from 1.13 g (7.5 mmol) 1-bromopentane to give 850 mg (93%) of **2a** as a white solid. M.p. 65 °C. ^1^H-NMR (d_6_-acetone): δ 0.88 (t, *J* = 7.3 Hz, 3 H, CH_3_), 1.32 (m, 6 H, 3 CH_2_), 1.83 (m, 2 H, CH_2_), 3.70 (s, 3 H, CH_3_), 4.62 (t, *J* = 6.7 Hz, 1 H, CH), 6.75 (s, 1 H, aromat. CH), 7.36 (s, 1 H, aromat. CH). ^13^C-NMR (d_6_-acetone): δ 14.3 (CH_3_), 23.3 (CH_2_), 26.6 (CH_2_), 31.9 (CH_2_) 31.9 (CH_3_), 32.4 (CH_2_), 65.1 (CH), 126.6 (aromat. CH), 135.6 (quart. C), 139.2 (aromat. CH). MS (CI) (m/z, %): 183 (M^+^+1, 90), 165 (100). MS (EI) (m/z, %): 182 (M^+^, 10), 111 (100). HR-MS Calcd. for C_10_H_18_N_2_O: 182.1419. Found: 182.1428. IR (KBr), ν, cm^−1^: 3262, 2951, 2854, 1508, 1416, 1237, 1072, 914.

### 1-(1-Methyl-1H-imidazol-5-yl)heptan-1-ol (2b)

The compound was prepared according “General Procedure 1” from 550 mg (5 mmol) 1-methyl-1*H*-imidazole-5-carbaldehyde (**1**) and the Grignard reagent prepared from 1.24 g (7.5 mmol) 1-bromohexane to give 820 mg (84%) of **2a** as a pale brown solid. M.p. 92 °C. ^1^H-NMR (d_4_-methanol): δ 0.90 (t, *J* = 6.8 Hz, 3 H, CH_3_), 1.32 (m, 8 H, 4 CH_2_), 1.86 (m, 2 H, CH_2_), 3.72 (s, 3 H, CH_3_), 4.64 (t, *J* = 6.9 Hz, 1 H, CH), 6.86 (s, 1 H, aromat. CH), 7.55 (s, 1 H, aromat. CH). ^13^C-NMR (d_4_-methanol): δ 14.4 (CH_3_), 23.7 (CH_2_), 27.1 (CH_2_), 30.2 (CH_2_), 32.2 (CH_3_), 33.0 (CH_2_), 36.8 (CH_2_), 65.5 (CH), 126.3 (aromat. CH), 136.1 (quart. C), 139.8 (aromat. CH). MS (CI) (m/z, %): 197 (M^+^+1, 100), 111 (21). HR-MS Calcd.: 196.1576. Found: 196.1580. IR (KBr), ν, cm^−1^: 3107, 2952, 1508, 1467, 1413, 1233, 1113, 1072, 1006, 930, 853, 825, 799, 701, 663.

### 1-(1-Methyl-1H-imidazol-5-yl)decan-1-ol (2c)

The compound was prepared according “General Procedure 1” from 550 mg (5 mmol) 1-methyl-1*H*-imidazole-5-carbaldehyde and the Grignard reagent prepared from 1.55 g (7.5 mmol) 1-bromononane to give 0.840 g (71%) of **2c** as a white solid. ^1^H-NMR (CDCl_3_): δ 0.88 (t, *J* = 6.7 Hz, 3 H, CH_3_), 1.27 (m, 14 H, 7 CH_2_), 1.86 (m, 2 H, CH_2_), 3.69 (s, 3 H, CH_3_), 4.61 (t, *J* = 6.9 Hz, 1 H, CH), 6.80 (s, 1 H, aromat. CH), 7.30 (s, 1 H, aromat. CH). ^13^C-NMR (CDCl_3_): δ 14.2 (CH_3_), 22.7 (CH_2_), 26.2 (CH_2_), 26.2 (CH_2_), 29.4 (CH_2_), 29.5 (CH_2_), 29.7 (CH_2_), 32.0 (CH_3_), 32.1 (CH_2_), 35.9 (CH_2_), 65.1 (CH), 126.3 (aromat. CH), 134.5 (quart. C), 138.7 (aromat. CH). MS (CI) (m/z, %): 239 (M^+^+1, 100), 111 (21). HR-MS Calcd. for C_14_H_26_N_2_O: 238.2045. Found: 238.2045. IR (KBr), ν, cm^−1^: 3265, 2921, 2852, 1512, 1473, 1083, 920, 668.

### 1-(1-Methyl-1H-imidazol-5-yl)undecan-1-ol (2d)

The compound was prepared according “General Procedure 1” from 550 mg (5 mmol) 1-methyl-1*H*-imidazole-5-carbaldehyde and the Grignard reagent prepared from 1.66 g (7.5 mmol) 1-bromodecane to give 658 mg (52%) of **2d** as a white solid. M.p. 97 °C. ^1^H-NMR (CDCl_3_): δ 0.88 (t, *J* = 7.0 Hz, 3 H, CH_3_), 1.27 (m, 16 H, 8 CH_2_), 1.89 (m, 2 H, CH_2_), 3.70 (s, 3 H, CH_3_), 4.63 (t, *J* = 7.1 Hz, 1 H, CH), 6.90 (s, 1 H, aromat. CH), 7.37 (s, 1 H, aromat. CH). ^13^C-NMR (d_4_-methanol): δ 14.1 (CH_3_), 22.7 (CH_2_), 26.1 (CH_2_), 29.3 (CH_2_), 29.4 (CH_2_), 29.5 (CH_2_), 29.6 (2 CH_2_), 31.9 (CH_2_), 31.9 (CH_3_), 35.7 (CH_2_), 65.4 (CH), 126.5 (aromat. CH), 134.1 (quart. C), 138.9 (aromat. CH). MS (EI) (m/z, %): 252 (M^+^, 17), 111 (100). HR-MS Calcd. for C_15_H_28_N_2_O: 252.2202. Found: 252.2199. IR (KBr), ν, cm^−1^: 3103, 2917, 2851, 1509, 1467, 1112, 1083, 823, 661.

### 1-(1-Methyl-1H-imidazol-5-yl)dodecan-1-ol (2e)

The compound was prepared according “General Procedure 1” from 550 mg (5 mmol) 1-methyl-1*H*-imidazole-5-carbaldehyde and the Grignard reagent prepared from 1.76 g (7.5 mmol) 1-bromoundecane to give 927 mg (70%) of **2e** as a white solid. M.p. 98 °C. ^1^H-NMR (CDCl_3_): δ 0.88 (t, *J* = 6.7 Hz, 3 H, CH_3_), 1.35 (m, 18 H, 9 CH_2_), 1.89 (m, 2 H, CH_2_), 3.70 (s, 3 H, CH_3_), 4.63 (t, *J* = 7.0 Hz, 1 H, CH), 6.87 (s, 1 H, aromat. CH), 7.36 (s, 1 H, aromat. CH). ^13^C-NMR (CDCl_3_): δ 14.1 (CH_3_), 22.7 (CH_2_), 26.1 (CH_2_), 29.3 (CH_2_), 29.4 (CH_2_), 29.5 (CH_2_), 29.6 (3 CH_2_), 31.9 (CH_2_), 31.9 (CH_3_), 35.7 (CH_2_), 65.2 (CH), 126.4 (aromat. CH), 134.1 (quart. C), 138.8 (aromat. CH). MS (EI) (m/z, %): 248 (M^+^ −18, 20), 219 (10), 135 (15), 121 (100), 108 (30). HR-MS Calcd. for C_16_H_30_N_2_O: 266.2358. Found: 266.2347. IR (KBr), ν, cm^−1^: 3269, 2918, 2851, 1513, 1472, 1236, 1111, 1089, 1068, 904, 668.

### 1-(1-Methyl-1H-imidazol-5-yl)tridecan-1-ol (2f)

The compound was prepared according “General Procedure 1” from 550 mg (5 mmol) 1-methyl-1*H*-imidazole-5-carbaldehyde and the Grignard reagent prepared from 1.87 g (7.5 mmol) 1-bromododecane to give 895 mg (64%) of **2f** as a white solid. M.p. 86 °C. ^1^H-NMR (d_4_-methanol): δ 0.90 (t, *J* = 7.0 Hz, 3 H, CH_3_), 1.32 (m, 16 H, 8 CH_2_), 1.86 (m, 2 H, CH_2_), 3.72 (s, 3 H, CH_3_), 4.64 (t, *J* = 7.0 Hz, 1 H, CH), 6.86 (s, 1 H, aromat. CH), 7.55 (s, 1 H, aromat. CH). ^13^C-NMR (d_4_-methanol): δ 14.4 (CH_3_), 23.8 (CH_2_), 27.2 (CH_2_), 30.5 (CH_2_), 30.6 (CH_2_), 30.7 (2 CH_2_), 30.8 (3 CH_2_), 32.2 (CH), 33.1 (CH_2_), 36.8 (CH_2_), 65.5 (CH), 126.3 (aromat. CH), 136.1 (quart. C), 139.8 (aromat. CH). MS (CI) (m/z, %): 281 (M^+^+1, 100), 263 (26). HR-MS Calcd. for C_17_H_32_N_2_O: 280.2515. Found: 280.2516. IR (KBr), ν, cm^−1^: 3103, 2921, 2850, 1510, 1470, 1270, 1111, 1070, 941, 823, 717, 664.

### 1-(1-Methyl-1H-imidazol-5-yl)-3-phenyl-propan-1-ol (2g)

The compound was prepared according “General Procedure 1” from 550 mg (5 mmol) 1-methyl-1*H*-imidazole-5-carbaldehyde and the Grignard reagent prepared from 1.39 g (7.5 mmol) 1-bromo-2-phenylethane to give 455 mg (42%) of **2g** as a viscous oil. ^1^H-NMR (CDCl_3_): δ 2.21 (m, 2 H, CH_2_), 2.75 (m, 1 H, CH_2_), 2.86 (m, 1 H, CH_2_), 4.62 (dd, *J* = 5.8 Hz, *J* = 8.2 Hz, 1 H, CH), 6.90 (s, 1 H, aromat. CH), 7.20 (m, 3 H, 3 aromat. CH), 7.28 (m, 2 H, 2 aromat. CH), 7.44 (s, 1 H, aromat. CH). ^13^C-NMR (CDCl_3_): δ 32.1 (CH_3_), 32.2 (CH_2_), 37.2 (CH_2_), 64.1 (CH), 125.4 (aromat. CH), 126.1 (aromat. CH), 128.5 (2 aromat. CH), 128.5 (2 aromat. CH), 134.2 (quart. C), 138.5 (aromat. CH), 141.2 (quart. C). MS (EI) (m/z, %): 216 (M^+^, 10), 111 (100), 83 (35). MS (CI) (m/z, %): 217 (M^+^+1, 100), 199 (23). HR-MS Calcd. for C_13_H_16_N_2_O: 216.1263. Found: 216.1262.

### 4-Chlorobenzoic acid 1-(1-methyl-1H-imidazol-5-yl)heptyl ester (3b)

The compound was prepared according “General Procedure 2” from 232 mg (1.18 mmol) **2b** and 964 mg (5.5 mmol) 4-chlorobenzoyl chloride to give 113 mg (29%) of **3b** as a pale yellow oil. ^1^H-NMR (CDCl_3_): δ 0.87 (t, *J* = 6.9 Hz, 3 H, CH_3_), 1.31 (m, 8 H, 4 CH_2_), 1.90 (m, 2 H, CH_2_), 6.10 (t, *J* = 7.5 Hz, 1 H, CH), 7.14 (s, 1 H, aromat. CH), 7.41 (s, 1 H, aromat. CH), 7.41 (d, *J* = 8.6 Hz, 2 H, 2 aromat. CH), 7.95 (d, *J* = 8.6 Hz, 2 H, 2 aromat. CH). ^13^C-NMR (CDCl_3_): δ 14.0 (CH_3_), 22.5 (CH_2_), 25.7 (CH_2_), 28.9 (CH_2_), 31.6 (CH_2_), 32.0 (CH_3_); 33.8 (CH_2_), 67.3 (CH), 1283 (quart. C), 128.8 (aromat. CH), 128.9 (2 aromat. CH); 130.6 (quart. C), 131.1 (2 aromat. CH); 138.9 (aromat. CH), 139.7 (quart. C). 165.2 (CO). MS (EI) (m/z, %): 335 (M^+^+1, 28), 239 (31), 179 (100). HR-MS Calcd. for C_18_H_23_ClN_2_O_2_: 334.1448. Found: 334.1450. IR (KBr), ν, cm^−1^: 2954, 2928, 2857, 1716, 1593, 1502, 1487, 1466, 1401, 1334, 1268, 1098, 1014, 760.

### 4-Chlorobenzoic acid 1-(1-methyl-1H-imidazol-5-yl)decyl ester (3c)

The compound was prepared according “General Procedure 2” from 235 mg (0.99 mmol) **2c** and 310 mg (1.8 mmol) 4-chlorobenzoyl chloride to give 90 mg (24%) of **3c** as a almost colourless oil. ^1^H-NMR (CDCl_3_): δ 0.84 (t, *J* = 7.2 Hz, 3 H, CH_3_), 1.21 (m, 14 H, 7 CH_2_), 2.06 (m, 2 H, CH_2_), 3.65 (s, 3 H, CH_3_), 6.07 (t, *J* = 7.2 Hz, 1 H, CH), 7.12 (s, 1 H, aromat. CH), 7.38 (d, *J* = 8.5 Hz, 2 H, 2 aromat. CH), 7.39 (s, 1 H, aromat. CH), 7.92 (d, *J* = 8.5 Hz, 2 H, 2 aromat. CH). ^13^C-NMR (CDCl_3_): δ 14.0 (CH_3_), 22.5 (CH_2_), 25.7 (CH_2_), 29.1 (2 CH_2_), 29.3 (2 CH_2_), 31.7 (CH_2_), 31.9 (CH_3_), 33.7 (CH_2_), 67.2 (CH), 128.2 (quart. C), 128.7 (2 aromat. CH), 128.8 (aromat. CH), 130.5 (quart. C), 130.9 (2 aromat. CH), 138.8 (quart. C), 139.6 (aromat. CH), 165.0 (CO). MS (EI) (m/z, %): 377 (M^+^+1, 70), 221 (100). HR-MS Calcd. for C_21_H_29_ClN_2_O_2_: 376.1918. Found: 376.1917. IR (KBr), ν, cm^−1^: 2926, 2854, 1716, 1594, 1496, 1458, 1398, 1275, 1171, 1095, 1017, 920, 852, 830, 767.

### Agar Diffusion Assay (DIN Method)

The bacteria and fungi were cultivated on AC agar (Sigma). The substances were placed on 6 mm paper discs on the agar, each impregnated with 50 μg of the tested compound or 50 μg of the reference drugs. The bacteria media were incubated for 24 h at 32 °C; the fungi media for 48 h at 28 °C, and the diameter of the zone of inhibition [mm] was then registered [15].

### Determination of MIC (DIN Method)

99 μL of a suspension of *Candida glabrata* (1 × 10^3^ CFU/ml) in All Culture media (AC Agar, Aldrich) were incubated with 1 μL ethanolic test solution for 36 h at 28 °C in a 96 well plate. After 36 h, the turbidity was measured at 590 nm and compared to the cell suspensions without the substance and AC agar media [15].

## Figures and Tables

**Sch. 1:**
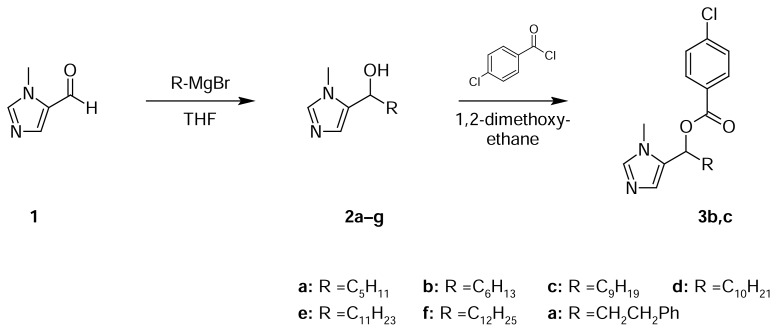
Synthetic preparation of the title compounds

**Tab. 1:** Agar diffusion assay (Te: tetracycline, Cl: clotrimazole, 50 μg/disc, inhibition diameter in [mm], 0: no inhibition)

	2a	2b	2c	2d	2e	2f	2g	3b	3c	Te	Cl
***Escherichia coli***	0	0	**0**	0	0	0	0	0	0	**30**	**0**
***Pseudomonas marginalis***	9	0	**15**	0	0	0	0	0	7	**28**	**15**
***Staphylococcus equorum***	15	0	**20**	**18**	**13**	10	0	7	0	**37**	**20**
***Streptococcus entericus***	0	0	7	**10**	8	7	0	7	0	**20**	**8**
***Candida glabrata***	0	0	**10**	**15**	9	7	0	**10**	6	0	**19**
***Aspergillus niger***	0	0	7	**11**	0	0	0	**0**	0	0	**15**
***Yarrowia lipolytica***	0	0	**13**	**11**	0	0	0	0	0	0	**22**
***Hyphopichia burtonii***	0	0	**15**	**16**	**10**	6	0	**12**	7	0	**23**
